# All That Glitters Isn't Gold: A Survey on Acknowledgment of Limitations in Biomedical Studies

**DOI:** 10.1371/journal.pone.0073623

**Published:** 2013-11-20

**Authors:** Gerben ter Riet, Paula Chesley, Alan G. Gross, Lara Siebeling, Patrick Muggensturm, Nadine Heller, Martin Umbehr, Daniela Vollenweider, Tsung Yu, Elie A. Akl, Lizzy Brewster, Olaf M. Dekkers, Ingrid Mühlhauser, Bernd Richter, Sonal Singh, Steven Goodman, Milo A. Puhan

**Affiliations:** 1 Department of General Practice, Academic Medical Center, University of Amsterdam, Amsterdam, The Netherlands; 2 Seminar für Sprachwissenschaft University of Tübingen, Tübingen, Germany; 3 Department of Communication Studies, University of Minnesota - Twin Cities, Minneapolis, Minnesota, United States of America; 4 Horten Centre for Patient-Oriented Research, University of Zurich, Zurich, Switzerland; 5 Ambulatorium Glattal, Zurich-Schwamendingen, Winterthur, Switzerland; 6 Department of Urology, University Hospital Zurich, Zurich, Switzerland; 7 Department of Internal Medicine, City Hospital Waid, Zurich, Switzerland; 8 Department of Epidemiology, Johns Hopkins Bloomberg School of Public Health, Baltimore, Maryland, United States of America; 9 Department of Internal Medicine, American University of Beirut, Beirut, Lebanon; 10 Department of Clinical Epidemiology and Biostatistics, McMaster University, Hamilton, Ontario, Canada; 11 Department of Medicine, State University of New York at Buffalo, Buffalo, New York, United States of America; 12 Departments of Internal and Vascular Medicine, Academic Medical Center, University of Amsterdam, Amsterdam, The Netherlands; 13 Department of Clinical Epidemiology, Leiden University Medical Centre, Leiden, The Netherlands; 14 Mathematics, Informatics, Natural Sciences Faculty, Health Sciences and Education, University Hamburg, Hamburg, Germany; 15 Institute of General Practice, University Hospital Düsseldorf, Heinrich-Heine University, Düsseldorf, Germany; 16 Division of General Internal Medicine, Johns Hopkins University, Baltimore, Maryland, United States of America; 17 Stanford University School of Medicine, Stanford, California, United States of America; 18 Institute for Social and Preventive Medicine, University of Zurich, Zurich, Switzerland; The Centre for Research and Technology, Hellas, Greece

## Abstract

**Background:**

Acknowledgment of all serious limitations to research evidence is important for patient care and scientific progress. Formal research on how biomedical authors acknowledge limitations is scarce.

**Objectives:**

To assess the extent to which limitations are acknowledged in biomedical publications explicitly, and implicitly by investigating the use of phrases that express uncertainty, so-called *hedges*; to assess the association between industry support and the extent of hedging.

**Design:**

We analyzed reporting of limitations and use of hedges in 300 biomedical publications published in 30 high and medium -ranked journals in 2007. Hedges were assessed using linguistic software that assigned weights between 1 and 5 to each expression of uncertainty.

**Results:**

Twenty-seven percent of publications (81/300) did not mention any limitations, while 73% acknowledged a median of 3 (range 1–8) limitations. Five percent mentioned a limitation in the abstract. After controlling for confounders, publications on industry-supported studies used significantly fewer hedges than publications not so supported (p = 0.028).

**Limitations:**

Detection and classification of limitations was – to some extent – subjective. The weighting scheme used by the hedging detection software has subjective elements.

**Conclusions:**

Reporting of limitations in biomedical publications is probably very incomplete. Transparent reporting of limitations may protect clinicians and guideline committees against overly confident beliefs and decisions and support scientific progress through better design, conduct or analysis of new studies.

## Introduction

Peer review has been defined as a “negotiation between author and journal about the scope of the knowledge claims that will ultimately appear in print” [1]. Surely, the acknowledgement of a study's limitations should be part of those negotiations. Goodman et al., in a study of the effect of peer review on manuscript quality, found that the acknowledgment of limitations was the most problematic item among 34 items of manuscript quality at submission [2]. Ioannidis, assessing 400 papers from six high-ranked scientific journals, found that often no limitations appeared to be mentioned [3]. Acknowledgement of limitations helps readers understand the compromises that had to be made in a study's design, perhaps due to budget constraints or other factors. Moreover, any discrepancies between the plans in the study protocol and the realities of study execution, such as participant non-adherence or incomplete data collection, may be highlighted. These imperfections of study execution, in particular, may otherwise be hard for readers to detect [4]. In 2002, Horton, noticing “the chaotic nature of discussion sections” in a sample of ten Lancet papers, wrote that the omission of limitations from the discussion sections must be judged “a potential failure of journal peer review” and he proposed a structured format for discussion sections [5].

The recognition that scientific papers often try to convince or influence readers [6], has led some scholars to study the linguistic and rhetorical aspects of scientific writing [7]. Horton even proposed “critical linguist analyses” as “a welcome third component” of peer review in addition to medical subject matter and statistical peer review [8].

Fletcher and Black discussing several ways in which “spin” occurs in the medical literature suggested that editors be more vigilant with industry-sponsored studies [9], while Yank et al, studying meta-analyses, found that for-profit funding was associated with more favorable conclusions, which were not accounted for by the results [10].

In this contribution, we studied how limitations are presented in biomedical research publications: *explicitly*, through an analysis of limitations sections, and *implicitly*, through linguistic analysis of the phrases used to moderate the strength of claims, also called “hedges” [11]. We hypothesized that industry-supported studies use fewer hedges in order to strengthen claims [9], [10], [12].

### Study objectives

In sub-study 1 we assessed (i) in what proportion of publications limitations are acknowledged, (ii) what types of limitations are discussed and (iii) in what manner, and (iv) whether the uncertainty arising from acknowledged limitations is reflected in the sections on implications for practice and conclusions. In sub-study 2 we analyzed the amount of uncertainty that is expressed and if that amount differed between journals, and industry-sponsored and other research in particular.

## Methods

### Sub-study on the number and nature of acknowledged limitations

From 10 general medical and 20 specialty journals, we included the first 10 publications describing randomized controlled trials (RCT), observational or diagnostic studies published in 2007. Within the group of medical and specialty journals, half were top journals (impact factor ranked 1^st^ through 5^th^); the other half ranked 11^th^ through 15^th^, according to journal impact factors (ISI 2007). Using a structured assessment form (web appendix S1), pairs of reviewers independently evaluated the proportion and type of acknowledged limitations and whether the conclusions in the abstract or discussion sections were tempered in light of limitations. Any disagreements between reviewers were resolved through discussion.

We assumed that 30% of publications would mention at least one limitation and calculated that we needed 300 papers to estimate that percentage with good precision (95% confidence interval ±5%). Results are reported using descriptive statistics. Logistic regression analysis was used to explore whether general medical and top ranking journals have different rates of acknowledged limitations than specialty journals.

### Sub-study to determine hedging scores

To determine hedging patterns, we used a software programme [13]. This program determines a “hedging score” for each sentence in a text. Hedges such as modal verbs (*may*, *could*), adverbs (*apparently*, *possibly*), and lexical verbs (*suggest [that]*, *believe [that]*) are detected, and each hedge is given a score between one and five, with higher scores reflecting more uncertainty [13]. The hedging score of a sentence is the sum of its individual hedging scores. To determine an article's hedging score, we summed all hedging scores for all sentences in the article. This score was then normalized by dividing it by the number of words in the title, abstract, introduction, methods, results, and discussion sections of that publication. We deemed industry support present (coded yes (1)/no (0)) if one of the following applied: full industry sponsorship of the research; (at least one) authorship by industry employees; industry donation of materials or lab space; industry payment for statistician-consultants; industry payment for administrative costs; industry payment for data collection; industry reviewing of a manuscript prior to submission.

The variable that coded for study quality was assigned the label ‘good (1)’ if at least one criterion applied, and as ‘less than good (0)’ otherwise, where randomized trials had to have concealment of randomization and/or proper random sequence generation, while observational studies were assessed for adjustment of confounding of the main association. Finally, the sample size was categorized in 6 categories using as cut-offs: 50; 100; 300; 1,000; 10,000. All data were extracted by one reviewer and checked for errors by another reviewer. Discrepancies were resolved through consensus. Three hundred publications were potentially eligible to measure the extent of hedging. We finally included 284 papers in the analyses of hedges. The ten papers from the journal *Clinical Gastroenterology and Hepatology* were missed in the transfer from our epidemiology team (MP, GtR) to the linguistic team that assessed the hedges (AG, PC) due to an administrative error that we detected only at the analysis stage. For six papers, the software program used to determine hedging patterns could not read the pdfs necessary to determine the hedging score.

For the sub-study on the hedging scores, we did not perform a sample size calculation, since we wanted to include as many of the papers from sub-study 1 as possible. We performed four multivariable linear regression analyses: two with the normalized hedging score, and two with the number of author-acknowledged limitations as the dependent variable, respectively [14], [15]. A dummy variable indicating industry support (coded as 1, 0 otherwise) was the independent variable of main interest. The other variables (RCT (yes (1) vs no (0)), study quality (high (1) vs non-high (0)), sample size (6 categories), p-value (3 categories) and journal (28 dummy variables for the 29 journals)) served to control for confounding. For the analyses on the numbers of limitations acknowledged and the hedging scores in turn, we first used a subset of 231 publications from which a meaningful p-value could be extracted (excluding, for example, prevalence surveys and diagnostic studies which report prevalence, and sensitivity and specificity of a test, respectively, which are not always accompanied by p-values). Next we analyzed 284 publications and controlled for *all* measured confounders. In all analyses we added variables to represent each of the 29 journals and controlled for any journal effect. The *New England Journal of Medicine (NEJM)* served as the reference category [14], [15]. In two sensitivity analyses, we assessed the stability of our results using the Huber-White sandwich estimator instead of the 28 dummies or used a random intercept model to account for the intra-journal clustering of papers which was caused by our method of sampling [16]. We visually checked the normality assumption using standardized normal probability plots. Stata 10.1 (College Station, TX, USA) software was used for all statistical analyses.

## Results

### Sub-study on the numbers and nature of acknowledged limitations

Eighty-one of the 300 publications did not acknowledge any limitation (27%, 95%CI from 22 to 32%). Two hundred nineteen publications acknowledged a median of 3 (range 1–8) limitations in the discussion section (73%, 68 to 78%), whereas 16 acknowledged a limitation in the abstract (5.3%, 3.3 to 8.5%). 186/300 (62%, 56 to 67%) of acknowledged limitations referred to aspects of internal validity, mostly to measurement errors (n = 149). 114/300 (38%, 33 to 44%) of acknowledged limitations referred to aspects of external validity, mostly to selected study populations (n = 115). 183/219 of the publications did not temper the conclusions because of limitations (84%, 78 to 88%). Publications in general medical journals were more likely to acknowledge limitations than publications in specialty journals (odds ratio 2.27, 95% CI from 1.27 to 4.10), particularly in abstracts (3.57, 1.27 to 10.0). Conclusions were not tempered more frequently in general medical journals. (0.98, 0.43 to 2.33). Journals' tier (rank 1–5 *vs* rank 11–15) did not affect these estimates.

### Sub-study on determinants of the hedging scores and numbers of acknowledged limitations

There were 61 (21.5%) industry-supported publications by our criteria. Table 1 shows that, on average, industry-supported publications were RCTs more often, were published in journals with higher impact factors, and had lower p-values. The proportion of good quality publications was similar in research with and without industry support. The interquartile range for the non-normalized weighted hedging score was from 86 to 160. If we assume an average hedging weight of three (the range was 1 to 5), this means that there were between 29 (86/3) and 54 (160/3) hedges with a weight of three in a publication with a length of about 3,255 (25^th^ centile) to 4,471 (75^th^ centile) words. Tables 2 and 3 show the results from the regression analyses. Hedging scores were lower (2.66 per 100 words) for industry-supported publications than for other publications (3.54), with a mean difference of 0.88 (95% CI 0.55 to 1.22). After controlling for confounding through multivariable linear regression analysis, this difference changed to 0.53 (0.06 to 1.00) for the set of 231 publications in which also a meaningful p-value could be extracted. The difference was 0.61 (0.15 to 1.07) for the full set of 284 publications, not controlling for the magnitude of the p-value.

**Table 1:** Descriptives of 284 publications from medium and top tier biomedical journals used to count and classify limitations and calculate the hedging scores.

	*No industry support*	*Industry support*	*Total*
*Characteristic*			
RCT, n (%)	33 (14.8)	47 (77.1)	80 (28.2)
Sample size	366 (100;1986)	313 (182;1520)	356 (103;1891)
Impact factor	5.78 (4.36;10.68)	10.68 (6.42;16.23)	6.36 (4.47;12.58)
p-value (n = 231)	.020 (.001;.050)	.011 (.001;.050)	.018 (.001;.050)
Quality good, n (%)	121 (54.3)	35 (57.4)	156 (54.9)
Top tier journal, n (%)	108 (48.4)	46 (75.4)	154 (54.2)
Raw score	124 (89;165)	101 (83;137)	116.5 (86;160)
No. words	3699 (3155;4341)	4195 (3608;5114)	3752 (3255;4471)
No. lines	168 (146;203)	204 (169;262)	175 (149;212)
Hedging score (%)	3.4 (2.6;4.3)	2.4 (1.8;3.2)	3.2 (2.4;4.2)
No. acknowledged limitations	0 (2;4)	0 (1;3)	0 (2;3.5)
Total, n (%)	223 (78.5)	61 (21.5)	284 (100)

Numbers are medians and (in brackets) interquartile ranges unless indicated otherwise; RCT = randomized controlled trial; Raw scores indicate the number of hedges in a publication (weighted by a hedging weight between 1 and 5); the hedging score is calculated by dividing the raw score by the number of words in (the relevant sections of) the publication. A hedging score of 3.0% indicates that on every 100 words there is one expression of uncertainty with a weight of 3 (or three with a hedging weight of 1, or less than 1, but with a hedging weight higher than 3, that is, expressing more uncertainty).

**Table 2:** Results of regression analyses for the hedging scores per 100 words.

	*Hedging score per 100 words*		
	*No industry support*	*Industry support*	*Difference*
Unadjusted (n = 284)	3.54 (3.37–3.71)	2.66 (2.37–2.94)	0.88 (0.55–1.22)
Fully adjusted (n = 231)			0.53 (0.06–1.00)
*-Journals*			
British Journal of Psychiatry			1.91 (0.43–3.40)†
British Medical Journal			1.46 (0.32–2.61)†
Annals of Family Medicine			1.92 (0.84–2.99)†
Not adjusted for P-value (n = 284)			0.61 (0.15–1.07)
*-Journals*			
Heart			1.31 (0.14–2.47)†
Pediatrics			1.22 (0.07–2.24)†
British Medical Journal			1.58 (0.44–2.71)†
Annals of Family Medicine			1.96 (0.88–3.04)†

† Journal differed significantly from New England Journal of Medicine (reference category).

Full adjustment was for Randomized Controlled Trial (yes/no), quality (high/non-high), sample size (6 categories), journal (28 dummies), magnitude of the P-value (3 categories).

**Table 3:** Results of regression analyses for the number of limitations acknowledged by authors.

	*Number of Limitations*		
	*No industry support*	*Industry support*	*Difference*
Unadjusted (n = 284)	2.32 (2.07–2.58)	1.49 (1.04–1.94)	0.83 (0.32–1.34)
Fully adjusted (n = 231)			0.65 (0.25–1.30)
*-Journals*			
American Journal of Medicine			1.58 (0.91–3.15)†
Annals of Internal Medicine			1.58 (0.06–3.10)†
Annals of Surgical Oncology			−2.18 (−3.81–−0.55)†
Chest			2.90 (1.27–4.53)†
Medicine			−2.61 (−4.87–−0.34)†
Not adjusted for P-value (n = 284)			0.64 (0.01–1.26)
*-Journals*			
American Journal of Medicine			1.77 (0.22–3.31)†
Annals of Family Medicine			1.47 (0.76–2.92)†
Annals of Internal Medicine			1.61 (0.13–3.10)†
Annals of Surgical Oncology			−1.97 (−3.49–−0.44)†
Chest			2.19 (0.68–3.70)†

† Journal differed significantly from New England Journal of Medicine (reference category).

Full adjustment was for Randomized Controlled Trial (yes/no), quality (high/non-high), sample size (6 categories), journal (28 dummies), magnitude of the P-value (3 categories).

Table 3 shows that, on average, industry-supported publications acknowledged 0.65 (0.25 to 1.30) fewer limitations than publications not supported by industry after full control for confounding.

Papers published in the British Journal of Psychiatry, the British Medical Journal and the Annals of Family Medicine used around 1.5 to 2 more hedges per 100 words than the NEJM. The publications from the Annals of Surgical Oncology acknowledged 2.18 fewer, Medicine 2.61 fewer, those from the American Journal of Medicine 1.58 more, Annals of Internal Medicine 1.73, and Chest 2.90 more limitations than those from the NEJM.

The results from the two sensitivity analyses were slightly less conservative than the results from the primary analyses presented here (data available on request).

## Discussion

We found that over a quarter of biomedical publications do not discuss any limitations. In abstracts, limitations are rarely mentioned. Industry-supported publications appear to express less uncertainty after controlling for factors that justify confidence in the study results. Finally, we found major differences between journals in how uncertainty is expressed and limitations acknowledged.

Complete reporting of study design, success of execution and, if appropriate, statistical analysis give meaning to the results of empirical studies. That is why, in science, we value (detailed) methods sections in research publications [4]. In addition, methods sections facilitate attempts at replication by others. It is often easier to *plan* a flawless study than to *execute* one. This distinction is important since readers can increasingly read the investigators' intentions through, for example, trial registration websites or separate publications dealing solely with a study's rationale, design and protocol details [17]. Access to details on study execution or log books is still rare [18]. In principle, one could place an unabridged list of all important differences between the study plan and actual execution in an appendix to a manuscript. However, deciding which events during a study's execution count as protocol violations and which violations cause limitations requires judgment [3]. Consider two examples: If the actual time interval between blood sampling and refrigerating the samples was 2 percent longer than stated in the protocol in 29 out of 1,000 samples collected for later determination of some compound, should one report that? By contrast, if in a randomized trial, a post-hoc chemical analysis shows that the interventional drug contained only 10% of the intended amount of the active compound, should that be acknowledged as a study limitation [19]? Our findings indicate that many authors appear not to be aware of limitations or are reluctant to admit them even after peer review.

Obviously, the present work would be quite incomplete if we did not address its limitations. First, for sub-study 1, the body of papers we assessed is five years old. However, we think that is unlikely that the awareness about acknowledging limitations has changed much, if at all, in recent years. Second, in sub-study 2, we lost 10, and could not analyze six of our 300 publication sample due to administrative errors or technical problems. Third, the software application used to calculate the hedging scores was not perfect. In particular, its accuracy is only about 93% [13]. Fourth, the weights assigned to the types of hedges are somewhat arbitrary. Fifth, it would have been interesting to determine an article's hedging score separately for each of the most relevant sections such as the title, abstract, results and discussion sections and thus be able to fine-tune the normalization using the word count, which was now summed across the whole publication. Sixth, the binary coding of industry support will have caused some misclassification. If misclassification were random, we may have underestimated the lower degree of hedging in industry-supported publications. A finer scale of industry support would have been attractive, but difficult to achieve.

As far as we are aware, research on the use of limitations is scarce. Previously, Ioannidis reported that 17% of publications mentioned any limitations. However, he used an automated search strategy on the texts that is likely to have missed acknowledgments of limitations [3]. Goodman et al., in a masked before-after study on manuscripts submitted to the Annals of Internal Medicine, found that newly submitted manuscripts scored worst on reporting about blinding, non-inclusions, drop-outs, multivariate methods, generalization of results and study limitations. Of those, reporting on limitations and generalization benefited most from peer review by physicians and epidemiologists trained in research methods, although even after peer review, the section on limitations remained among the weaker sections [2]. Our work does not shed light on the question of why industry support should lead to the expression of less uncertainty, all other factors being equal? However, there is a vast field specialized in writing texts for the industry and Carl Elliott wrote on the more cynical aspects thereof [20]. More recently, Medtronic, a US medical device company, has been accused of manipulating 13 journal articles and paying large amounts of money to authors [21].

Although the STROBE statement encourages authors to “Discuss limitations [..], taking into account sources of potential bias or imprecision. Discuss both direction and magnitude of any potential bias”, “Give a cautious overall interpretation of results considering objectives, limitations, [..] etc.” and the CONSORT statement urges authors to address “Trial limitations, addressing sources of potential bias, imprecision, and, if relevant, multiplicity of analyses.” [22], [23], it will probably not be easy to curb the tendency of many authors to only sparingly admit issues that were imperfect by design, execution or both. Until a major change in attitudes occurs, ways to improve the reporting of all serious limitations in biomedical research reports may require a structured approach at the editorial offices: Explicit journals' instructions for authors, more structured support and dedicated checklists for peer reviewers, more structured approaches at the editorial offices, perhaps involving automatic comparison of a manuscript's hedging score to reference values for hedging scores conditional on the study's design and outcome. Finally, checks on the number and nature of limitations acknowledged and if acknowledged limitations are reflected in the strength of the conclusions drawn may be useful. After publication, web-based rapid response facilities and acknowledgment for those critics who enlighten readers as to serious limitations may be considered. On the other hand, the authors may sometimes be the only ones who know about a limitation due to problems during study execution and have to decide whether it is important enough to be mentioned as a limitation. We believe that in general, editors may encourage authors to write about their methods used more extensively at the expense of the length of the discussion sections that are sometimes filled with much speculation. Spending at least a fixed proportion of the discussion section on limitations may also be explored [5]. We would also welcome if more journals followed the Annals of Internal Medicine's rule that the abstract should mention at least one limitation. In the meantime perhaps the National Library of Medicine may consider adding limitations to the abstracts in PubMed.

Replication of this work is needed. Our work may be improved by calculating hedging scores per section. Sensitivity analyses with the hedging-weights may shed light on the role of the weights assignment in the hedging software. Some of the ideas we suggested above may be carried out as research projects at the editorial offices. For example, we may try to assess if more structured approaches are implementable, what the challenges involved are with such approaches, and what effects can be measured after implementation on for example transparent reporting, time investment needed, authors avoiding journals with a strict approach in place, and effects on journals' impact factors. Long-term effects may involve the quality of clinical practice guidelines and patient care and its outcomes. And finally, larger studies may try to replicate and investigate in depth how by-journal hedging score variation is related to editorial policies.

In conclusion, our data show that reporting of limitations to original biomedical research is probably incomplete. Either directly, through clinical decision-making by evidence-based clinicians or indirectly through its effects on systematic reviews and clinical guidelines, optimal patient care may be jeopardized. Finally, scientific progress may be slowed down: reporting limitations more completely would aid the design and implementation of future studies. An appropriate amount of hedging given these limitations could further guide future scientific inquiry.

## Supporting Information

Appendix S1 **Data extraction form for sub-study 1 (n = 300).**
(DOC)
