# Supplementary material for: All That Glitters Isn't Gold: A Survey on Acknowledgment of Limitations in Biomedical Studies
Source: PLoS One. 2013 Nov 20;8(11):e73623. doi: 10.1371/journal.pone.0073623 (PMC3854521; doi:10.1371/journal.pone.0073623)
Supplement: Appendix S1 — Data extraction form for sub-study 1 (n = 300). (DOC) [file pone.0073623.s001.doc]

Appendix Form S1. Data extraction form for sub-study 1 (n=300)

date: study identification number: author: reviewer:

**Acknowledgement of limitations**

1. **Quantity of limitations**
   1. Are limitations reported in the **entire paper** at all?

yes

no

- 1. Are limitations mentioned in the **abstract**?

Yes, anywhere

yes, in specific section

no

- 1. Is there a specific paragraph (with or without subheading) for limitations in the **discussion section**?

yes

no, go to 1.5

1.4 Count number of paragraphs and divide into thirds. Where is the limitation section placed in the **discussion**?

at the beginning of discussion (in first third of paragraphs)

in the middle of discussion (in second third of paragraphs)

at the end of discussion (in last third of paragraphs)

1.5 If there is no specific limitation section but if limitations are mentioned somewhere in the text, where are they acknowledged?

at the beginning of discussion (in first third of paragraphs)

in the middle of discussion (in second third of paragraphs)

at the end of discussion (in last third of paragraphs)

1. **Quality of limitations**

Mark type of limitation for each limitation reported in the article using the table below.

1. **Countering limitations**

Did the authors counter the limitation? If yes did they justify themselves or turn it into a strength? Answer to these questions for each limitation using the table.

- May the result (point estimate) be influenced be the limitation? 🡪 internal validity
- Is the study setting different from the daily practice setting? 🡪 external validity

|  | Limitations (sequentially) | 1 | 2 | 3 | 4 | 5 | 6 | Counter of limitation?^#^ | | |
| --- | --- | --- | --- | --- | --- | --- | --- | --- | --- | --- |
|  |  |  |  |  |  |  |  | Tempering of importance | | Conversion into strength |
| **applies to** | **Aspects of internal validity** | | | | | | | | | |
| RCT, Obs | Control for confounding |  |  |  |  |  |  |  | |  |
| All | Consecutive patients |  |  |  |  |  |  |  | |  |
| RCT | Masking patients |  |  |  |  |  |  |  | |  |
| RCT, Obs | Masking outcome assessors |  |  |  |  |  |  |  | |  |
| RCT | Masking health care providers |  |  |  |  |  |  |  | |  |
| All | Measurement/ misclassification error of exposures /outcomes /reference standard |  |  |  |  |  |  |  | |  |
| All | No-pre-specified analyses (e.g. subgroup) |  |  |  |  |  |  |  | |  |
| RCT | Treatment protocol adherence |  |  |  |  |  |  |  | |  |
| RCT, Obs | Loss to follow-up |  |  |  |  |  |  |  | |  |
| RCT, Obs | Missing data |  |  |  |  |  |  |  | |  |
| All | Limitation unclear |  |  |  |  |  |  |  | |  |
| All | Other: (e.g. variable not measured) |  |  |  |  |  |  |  | |  |
|  | **Aspects of external validity** | | | | | | | | | |
| All | Study participants (e.g. patients) |  |  |  |  |  |  |  |  | |
| All | Exposures/treatments/Dx tests |  |  |  |  |  |  |  |  | |
| All | Outcomes |  |  |  |  |  |  |  |  | |
| All | Sample size small/power too low |  |  |  |  |  |  |  |  | |
| All | Other: |  |  |  |  |  |  |  |  | |

RCT: Randomised controlled trials; Obs: Observational studies; Dx: diagnostic studies

^#^ leave blank if “no”

1. **Relation between limitations and conclusion**

4.1 In the discussion section, does the acknowledgment of limitation(s) come before a conclusion or implications for practice?

(For example, if the first paragraph of the discussion contains a conclusion or implications for practice and limitations are acknowledged later, the answer would be “no”)

yes

no

4.2 If limitations are acknowledged, do the conclusions or implications for practice reflect the uncertainty that arises from the limitations?

yes

no

1. **Industry-sponsored study**

Yes

No or not stated

1. **Comments (on any aspects of acknowledgement of limitations not covered above)**
